# Vitamin D gene polymorphisms are associated with type 1 diabetes mellitus in Korean youth

**DOI:** 10.1186/1687-9856-2013-S1-P24

**Published:** 2013-10-03

**Authors:** Hyo-Kyoung Nam, Young Jun Rhie, Kee-Hyoung Lee

**Affiliations:** 1 Department of Pediatrics, College of Medicine, Korea University, Seoul, Republic of Korea

## 

Vitamin D deficiency is reported in patients with type 1 diabetes mellitus (T1DM). Allelic variations in the gene associated with vitamin D metabolism were reported to play a role in glucose metabolism. CYP2R1 and CYP27B1 gene polymorphisms were investigated as a candidate gene for T1DM in Korean youth.

We measured serum level of 25-hydroxyvitamin D and 1,25-dihydroxyvitamin D in 217 subjects and defined vitamin D deficiency as 25-hydroxyvitamin D level below 20 ng/ml. Five single nucleotide polymorphisms (SNPs) in the CYP2R1 gene and three SNPs in the CYP27B1 gene were examined in 92 patients with T1DM and 125 controls. The frequency of A/G alleles of the rs12794714 was 35.9%/64.1% and 46.0%/54.0% in T1DM youth and controls, respectively (p=0.034). The G/A alleles of the rs10766196 was 37.0%/63.0% and 46.0%/54.0% in T1DM youth and controls, respectively (p=0.059). We observed that the GG homozygous genotype of rs12794714 and AA homozygous genotype of rs10766196 were more prevalent in T1DM youth than in controls (42.4% vs. 29.6%, p=0.051 and 41.3% vs. 29.6%, p=0.073, respectively). The prevalence of vitamin D deficiency was considerably higher in T1DM youth with ‘G’ allele of rs12794714 and ‘A’ allele of rs12794714.

Polymorphisms in CYP2R1 gene are associated with susceptibility to T1DM in Korean youth. The pathophysiological mechanisms remain unexplained, but they could be related to an etiological role for vitamin D deficiency in T1DM.

